# How do chemical epistemological beliefs affect Chinese students' chemistry disciplinary competence? A structural equation modeling analysis

**DOI:** 10.3389/fpsyg.2025.1599442

**Published:** 2025-11-11

**Authors:** Xiujin Zhu, Yanning Huang, Hailiang Liu

**Affiliations:** 1 College of Teacher Education, Capital Normal University, Beijing, China; 2 School of Education, Trinity College Dublin, Dublin, Ireland

**Keywords:** chemical epistemological beliefs, chemistry disciplinary competence, critical thinking disposition, chemistry learning approaches, structural equation modeling

## Abstract

The shift in focus from knowledge dissemination to the cultivation of disciplinary competence has become a pivotal topic in K-12 science education reform. Scientific epistemological beliefs play a crucial role in students' learning processes and overall development. Although chemistry represents a vital component of science, little is known about how chemical epistemological beliefs affect students' chemistry disciplinary competence. This study aimed to explore the mechanisms by which epistemological beliefs influence students' chemistry disciplinary competence and to provide suggestions for fostering it. A sample of 182 11th-grade students participated in the formal evaluation. The results showed that (1) among the four dimensions of chemical epistemological beliefs—Source, Certainty, Justification, and Simplicity—the source and certainty beliefs substantially influenced chemistry disciplinary competence; (2) critical thinking disposition and chemistry learning approaches played separate mediating role in the influence of chemical epistemological beliefs on competence, and (3) the chain mediating effects of chemical epistemological beliefs, critical thinking disposition, chemistry learning approaches, and chemistry disciplinary competence were also significant. Findings suggest that developing chemistry disciplinary competence requires fostering sophisticated chemical epistemological beliefs to cultivate critical thinking disposition and deep learning approaches.

## 1 Introduction

Epistemological beliefs refer to individual beliefs concerning the nature of knowledge (i.e., what knowledge is) and the nature of knowing (Hofer and Pintrich, 1997). Numerous studies have examined the relationship between epistemological beliefs and student learning outcomes (Acar, 2019; Hofer and Pintrich, 1997; Özbay and Köksal, 2021; Schommer-Aikins and Easter, 2006; Tolhurst, 2007; Trautwein and Lüdtke, 2007; Youn et al., 2001; Wang H.-H. et al., 2022). However, representing learning outcomes solely through grade point average (GPA) may inadequately capture the development of students' specific abilities.

Disciplinary competence has recently become a focal point in international K-12 education. Science curriculum reforms worldwide have shifted their focus from assessing students' knowledge acquisition to evaluating their disciplinary competence. According to the Next Generation Science Standards, students should possess robust science-based skills, not only within specific content areas but also in critical thinking (CT) and inquiry-based problem solving. Assessments should aim to meet performance expectations by enhancing student knowledge and competence (National Research Council, 2012). The Chinese senior high school chemistry curriculum standard also specifies the key competencies that students are expected to develop through chemistry courses, and formulates a clear framework for evaluating chemistry disciplinary competence (CDC; Wang and Wei, 2018). Although the relationship between epistemological beliefs and student learning outcomes was tested in early research, given that GPA does not clearly represent students' abilities, and few studies have explored the relationship between epistemological beliefs and specific science-related competence (Schiefer et al., 2022). Our work primarily contributes to using the chemistry disciplinary competence framework to replace GPA for a more comprehensive and accurate representation of students' ability development after chemistry learning, and to exploring which dimensions of chemical epistemological beliefs influence the development of students' chemistry disciplinary competence. Since prior research suggests that critical thinking (CT) disposition and learning approaches are shaped by epistemological beliefs and contribute to students' academic performance. This study also investigates whether CT disposition and chemistry-specific learning approaches mediate the relationship between students' epistemological beliefs about chemistry and their development of chemistry disciplinary competence.

## 2 Literature review

### 2.1 Chemical epistemological beliefs and chemistry disciplinary competence

In this part, at first, we introduce the theoretical evolution and dimensional development of epistemological beliefs. Based on interviews and surveys with Harvard students, Perry's (1968) research pioneered the psychological study of epistemological beliefs and examined how individuals' views on knowledge have evolved. He outlined four progressive stages in the development of students' personal epistemology: dualism, multiplism, relativism, and a committed form of relativism. Based on Perry's research, Schommer (1990) proposed an epistemological belief system comprising five independent dimensions: structure of knowledge, certainty of knowledge, source of knowledge, control of knowing, and speed of knowledge acquisition. Hofer and Pintrich's (1997) review synthesized research on personal epistemology into an “epistemological theory” model, identifying two core aspects of epistemological beliefs: the nature of knowing (certainty and simplicity) and the nature of knowledge (source and justification). This four-dimensional framework has been widely applied in education to explore the link between epistemic beliefs and learning outcomes (Ding, 2014). As research on epistemological beliefs has deepened, the role of epistemological beliefs within specific disciplines has gained increasing emphasis. Aditomo (2018) suggested that the relationship between epistemological beliefs and academic performance may vary across disciplines. Scientific epistemological beliefs are crucial to understanding scientific concepts (Liang and Tsai, 2010; Tsai et al., 2011). Previous research has highlighted the role of epistemological beliefs in science; however, further exploration within specific disciplines, such as biology and chemistry, is necessary (Lin et al., 2012). To fill this research gap in chemistry, the present study constructs the framework of chemical epistemological beliefs based on the four-dimensional model proposed by Hofer and Pintrich (1997) and the disciplinary characteristics of chemistry. These dimensions are defined as follows. Source of chemistry knowledge: Beliefs about whether chemical knowledge is constructed through inquiry and experimentation or transmitted by authority; Certainty of chemistry knowledge: Beliefs about whether chemical knowledge is fixed or evolving. Sophisticated beliefs view chemical concepts as open to revision based on new evidence; Simplicity of chemistry knowledge: Beliefs about whether chemistry is a set of isolated facts or an integrated system of interrelated concepts; Justification for chemistry knowing: Beliefs about how chemical claims are validated whether through evidence and reasoning, or accepted based on authority or intuition. These adapted definitions clarify how students perceive the nature of chemical knowledge and serve as the conceptual foundation for this study.

Against the backdrop of shifting international education reform focus from knowledge acquisition to competence development, traditional academic assessment methods—such as grade point average (GPA) and conventional tests—are increasingly recognized as insufficient for capturing the complexity of students' development in chemistry. In the 1960s, research on disciplinary competence began to attract academic attention. Bloom et al.'s (1956) work on the taxonomy of educational objectives formed the basis for the assessment of disciplinary competence, with a focus on cognitive development in areas such as knowledge, comprehension, analysis, synthesis, and evaluation. Biggs and Collis's (1982) Structure of the Observed Learning Outcome (SOLO) Taxonomy outlines five levels of cognitive development, ranging from prestructural understanding to extended abstract thinking. Compared to Bloom et al.'s focus on internal cognitive processes, the SOLO Taxonomy theory emphasizes observable student outcomes, specifically regarding their disciplinary competence. In the field of science education, The Next Generation Science Standards (NGSS) outline a progression of cognitive demands, which reflects increasing cognitive complexity in science learning and assessment. This progression encompasses three levels: routine production, where students demonstrate core ideas in familiar contexts; typical application, where knowledge and practices are integrated to solve standard problems; and addressing a high degree of uncertainty, where students synthesize scientific, engineering, and crosscutting concepts to engage with complex or novel situations. Embedded within the NGSS Performance Expectations, this design moves students from basic applications of disciplinary knowledge toward higher-order reasoning and problem-solving, thereby establishing an evaluative framework that anticipates progressively more sophisticated cognitive engagement (National Research Council (NRC), 2013). Within the chemistry discipline, Zhang et al. (2021) based on Bloom's Taxonomy and analysis of international science assessments (PISA, TIMSS, NAEP) developed a chemistry task classification system. It has three dimensions: Concept-Knowing (e.g., “Recognize” organic functional groups, “Represent” chemical equations); Concept-Application (e.g., “Compare” exothermic/endothermic reactions, “Explain” gas solubility); Problem-Solving (e.g., “Predict” concentration's impact on reaction rate, “Design” acid molar mass experiments). Talanquer (2021) pointed out that Multifaceted Chemical Thinking is a core competence of students and proposed six aspects: Granularity, Dimension, Frame, Basis, Mode, and Focus. Among them, Granularity, Dimension, and Frame are more of the cognitive perspectives for students to understand chemistry, while Basis, Mode, and Focus are the competence levels required for students to solve chemical problems, mainly including understanding, explanation, application, reasoning, and so on. These framework link theoretical models to chemistry assessment. Building on prior research, Wang L. et al. (2022) defined chemistry disciplinary competence (CDC) based on the evolving focus of international science education reforms and disciplinary competence frameworks. Specifically, this competence is referred to as “the stable psychological regulatory mechanism required for students to engage in cognitive and problem-solving activities when facing chemistry research objects and problem contexts, manifested in their ability to complete tasks such as understanding, application, transfer, and innovation using knowledge and cognitive modes.” On the basis of this definition, they further developed a framework to assess high school students' chemistry disciplinary competence, which focuses on three cognitive dimensions: Understanding, Applying, and Innovating. This conceptualization shows strong structural consistency with the PISA 2025 science literacy framework, which similarly identifies three core scientific competencies: explaining phenomena scientifically, constructing and evaluating designs for enquiry and interpreting data and evidence critically, and researching and using scientific information for decision-making and action (OECD, 2023). Both frameworks adopt a tiered progression from foundational understanding, to application in inquiry and problem-solving contexts, and ultimately to higher-order innovation or decision-making in complex situations. Given this alignment, the present study employs Wang's CDC framework as an operationalization of disciplinary competence in chemistry, ensuring both theoretical grounding in international science education standards and disciplinary specificity to chemistry learning. The task and performance items used to assess students' chemistry disciplinary competence are shown in Table 1. Responses to these tasks are a measure of students' academic performance and can be used to outline their level of cognitive development. This framework has been applied in the evaluation of the new round of China's chemistry curriculum reform. Moreover, some scholars have used this framework to assess the development of students' chemistry disciplinary competence after project-based learning, and found that it can effectively reflect students' overall performance in chemistry competence (Zhao and Wang, 2022).

**Table 1:** Task and academic performance items to test chemistry disciplinary competence.

**Competence**	**Task**	**Academic performance**
Understanding	Recognize	Remember the information and extract relevant knowledge from known information. (A1)
	Generalizing	Organize knowledge by identifying and summarizing its core attributes, and clarify the connections among related concepts and principles. (A2)
	Illustrating	Integrate existing knowledge and experience to explain and validate target knowledge. (A3)
Applying	Explaining	Use material properties and principles to analyze, explain phenomena, and assess the rationality of similar activities. (B1)
	Predicting	Utilize material properties and principles to predict outcomes, draw conclusions from evidence that address the problem or hypothesis, and perform related tasks. (B2)
	Designing	Execute experiments based on experience prototypes to answer questions or test hypotheses, selecting optimal designs and tools. (B3)
Innovating	Complex Reasoning	Apply core material knowledge to solve complex, multifaceted problems through systematic reasoning. (C1)
	Systematic Investigating	Perform remote migration of prototypes, systematically explore scientific problems, and complete tasks from developing hypothesis to providing evidence. (C2)
	Creative Thinking	Integrate diverse knowledge to derive new conclusions, apply imaginative creativity to material properties, and design innovative solutions for complex problems. (C3)

Chemistry disciplinary competence does not deny the importance of knowledge; instead, it extends beyond mere mastery of symbolic knowledge, emphasizing the transformation of subject knowledge into disciplinary competence (Guo and Ma, 2012). Disciplinary competence development is still based on knowledge, not only as a noun (knowledge) but also as a verb (knowing). Scientific epistemological beliefs play a crucial role in understanding and interpreting scientific knowledge (Elder, 1999; Lederman, 1992). Therefore, cognitive processing of chemistry disciplinary competence is contingent upon the prior development of chemical epistemological beliefs. Despite established links between general epistemology and competence, chemistry education research faces gaps: (1) The theoretically established dimensions of chemical epistemological beliefs lack empirical validation regarding their differential effects on the CDC; (2) Potential chemistry-specific mediating mechanisms (such as metacognition or critical thinking) through which epistemological beliefs enable the transformation of knowledge into competence remain largely unexamined. Given this conceptualization of chemistry disciplinary competence as an integrated capacity, it becomes critical to understand the psychological precursors that facilitate its development, particularly domain-specific epistemological beliefs about the nature of chemical knowledge and knowing.

### 2.2 Potential mediating effect of CT disposition

CT is a multifaceted construct with various definitions (Moore, 2013); however, most of its definitions acknowledge two main aspects—the cognitive (CT skills) and dispositional (CT disposition) dimensions (Facione et al., 2000a; Lai, 2011; Manassero-Mas et al., 2022). CT disposition emphasizes the social-emotional aspects of CT, that is, the effect on one's willingness to use CT skills and self-monitor how well they are performed (Ren et al., 2020). In other words, CT disposition refers to an individual's internal motivation to engage in CT when addressing problems, evaluating ideas, or making decisions (Chen et al., 2024). However, the theoretical link between students' epistemological beliefs and their disposition toward critical thinking (CT) remains underexplored. Epistemological orientation is essential for CT (McGinnis, 2016), and epistemological beliefs influence how individuals handle and apply knowledge, shaping their thinking, learning, and motivation (Schraw and Sinatra, 2004). Sophisticated epistemological beliefs underlie flexible thinking, which is essential for CT (N.-M. Chan et al., 2011). Additionally, epistemological beliefs significantly predict CT disposition (Orhan, 2022). Therefore, they serve as predictors of CT. CT disposition influences subject competency through diversified mediation mechanisms. Phan (2009) showed enhanced academic achievement when students fully utilized CT. For a sample of Chinese students, Liu et al. (2023) also found that students with a CT orientation were more likely to analyze and solve problems rationally, leveraging abilities that significantly enhanced their academic success.

Despite these established connections between epistemological beliefs, CT disposition, and academic outcomes (including subject competence), significant research gaps persist in understanding the interplay between chemical epistemological beliefs, CT disposition, and chemistry disciplinary competence. First, epistemological beliefs predict general CT disposition domain-specific pathways linking chemical epistemological beliefs dimensions to chemistry-contextualized CT disposition remain unexamined. Second, the differential mediation effects of CT disposition sub-facets on chemistry disciplinary competence components lack empirical validation. Third, contextual variations in mediation necessity across competence types are overlooked. These limitations necessitate domain-specific models examining potential pathways in chemistry education.

### 2.3 Potential mediating effect of chemistry learning approaches

Learning approaches are strategies learners use to engage in academic tasks, which subsequently affect their learning outcomes (Li et al., 2013), and are of two types: deep learning and surface learning (Biggs, 1987; Chin and Brown, 2000). Students who perceive learning as a process of transforming information tend to deploy a deep learning approach that focuses on understanding the meaning of the material. Contrarily, students who view learning as reproducing knowledge tend to deploy a surface learning approach that emphasizes the reproduction of those materials (Rodríguez and Cano, 2006). Consequently, epistemological beliefs are likely to be a prerequisite for students' chosen learning approach. It has been demonstrated that epistemological beliefs impact students' learning approach (Chan, 2003; Rodríguez and Cano, 2007).

By extension, students who employ deep learning strategies are more inclined to demonstrate a high-level of disciplinary competence. They aim to understand new concepts for personal growth and appreciate the educational purpose of their tasks. Conversely, those using a surface approach concentrate on fulfilling immediate assignment requirements, potentially misinterpreting a task's deeper intent (Bliuc et al., 2011). Therefore, chemistry learning approaches are likely a predictor of chemistry disciplinary competence. However, the empirical validation of this mediated pathway remains unexplored.

### 2.4 Mediating effect of CT disposition and chemistry learning approaches

Finally, we need to clarify the relationship between CT disposition and chemistry learning approaches. CT disposition predicts individuals' actions or reactions in various situations (Facione et al., 2000a). In other words, a positive CT disposition is a prerequisite for students adopting deep learning methods. Students with CT disposition tend to collect various types of information while studying, indicating a preference for deep learning approaches (Pu et al., 2019). Thus, CT disposition may have a positive impact on chemistry learning approaches.

## 3 The theoretical framework and research hypotheses

The above literature review shows that epistemological beliefs will affect the cognitive process of student learning and ultimately affect student disciplinary competence. While both epistemological beliefs and disciplinary competence are domain-specific (Guo and Ma, 2012; Lin et al., 2012), no studies were located that examined the relationship between chemical epistemological beliefs and chemistry disciplinary competence. The primary objective of this research is to investigate how chemical epistemological beliefs influence the progression of students' chemistry disciplinary competence.

Furthermore, each dimension of epistemological belief operates independently, as a student may have strong beliefs in one dimension but weak beliefs in another (Schommer, 1990). Different dimensions of epistemological beliefs have varying impacts on students' academic performance (Kizilgunes et al., 2009). Studies of epistemological beliefs and students' academic performance have paid little attention to the epistemological beliefs that affect the development of students' specific abilities. Therefore, we aim to investigate which dimensions of chemistry epistemological beliefs influence students' chemistry disciplinary competence performance.

Accordingly, this study explores the relationship between chemistry epistemological beliefs and the development of high school students' chemistry disciplinary competence. Given that epistemological beliefs can enhance students' CT disposition, the increase in CT disposition is conducive to promoting students' academic performance. It could be hypothesized that chemistry epistemological beliefs can influence students' chemistry disciplinary competence through CT disposition. This study hypothesizes that the impact of chemistry epistemological beliefs on chemistry disciplinary competence is mediated by CT disposition.

Given that epistemological beliefs could promote students' learning approaches, which is an important condition for developing disciplinary competence, we assume that chemistry learning approaches also play a mediating role in the relationship between chemistry epistemological beliefs and chemistry disciplinary competence. Taking this one step further, as both CT disposition and learning approaches may mediate the effects of chemistry epistemological beliefs on chemistry disciplinary competence, we assume that chemistry epistemological beliefs influence chemistry disciplinary competence through the chain-mediating effect of chemistry CT disposition and chemistry learning approaches.

Guided by the preceding analysis, the research hypotheses are formulated as follows:

Hypothesis 1: Students' chemical epistemological beliefs significantly predict their chemistry disciplinary competence in high school contexts.

Hypothesis 2: CT disposition mediates the association between chemical epistemological beliefs and chemistry disciplinary competence.

Hypothesis 3: Chemistry learning approaches act as a mediator, transmitting the effects of chemical epistemological beliefs to chemistry disciplinary competence.

Hypothesis 4: A chain-mediating mechanism links chemical epistemological beliefs to chemistry disciplinary competence, with CT disposition and Chemistry learning approaches transmitting the effects in sequence.

**Figure 1:**
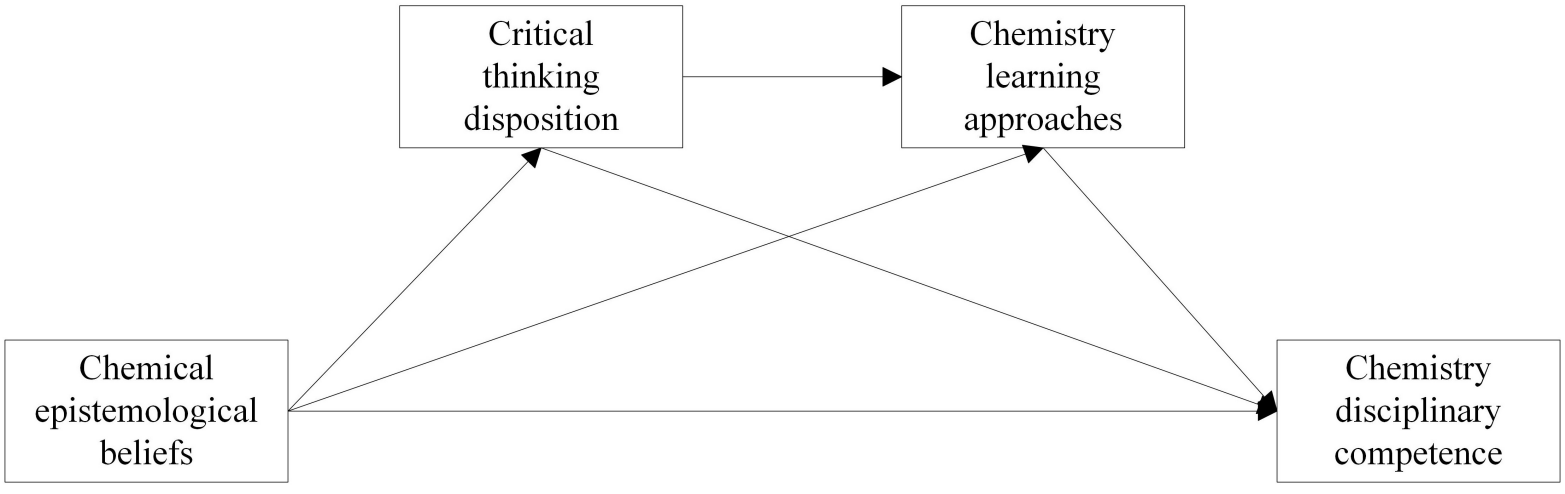
Conceptual model.

The conceptual model constructed in this study is shown in Figure 1.

## 4 Methods

### 4.1 Participants

After a 96-person pilot study to verify the reliability and validity of the tool and to revise the tool, a total of 182 students (52% male, 48% female) in the 11th grade from four high schools in eastern China participated in formal research. The schools represented model public high schools and ordinary high schools, and all students were from regular classes. Participants' ages ranged from 16 to 18 years, with an average age of 17 years. All students selected chemistry as their major subject and completed the high school organic chemistry module. The school principals, teachers, and parents provided consent to participate in the study. Students voluntarily participated in the study, and their data were kept confidential. Invalid samples (e.g., those with one or more unanswered items) were excluded from data analysis.

### 4.2 Survey instruments

The measures of chemical epistemological beliefs, CT disposition, and chemistry learning approaches were derived from established English questionnaires. To ensure readability, all items were translated into Chinese, and two science education experts reviewed and refined the content for accuracy. In China, the formulation of college entrance examination questions takes curriculum standards as the fundamental basis: the curriculum standards have very specific regulations on students' output-oriented proficiency levels after they complete chemistry knowledge learning, and these regulations are mainly clearly reflected in the “Academic Requirements” section. For example, the action-oriented requirements such as “identify, predict, analyze, explain, illustrate, judge, and describe” mentioned in the “Academic Requirements” clearly point to the types of tasks that students should be able to complete after learning the corresponding knowledge, and these task types exactly constitute an important basis for the assessment framework of chemistry disciplinary competence (Yu, 2018). The measures for assessing students' chemistry disciplinary competence were adapted from college entrance examination questions. The validity of the measures of students' chemistry disciplinary competence was established by an expert review combined with the Rasch measurement.

#### 4.2.1 Chemical epistemological beliefs

The instrument used to measure chemical epistemological beliefs was adapted from the General Epistemological Beliefs Questionnaire (Hofer, 2000). Twenty items were selected and adapted according to chemistry characteristics to form the Chemical Epistemological Beliefs Questionnaire (CEBQ). All items were presented on a Likert-type scale ranging from 1 (strongly disagree) to 7 (strongly agree). In the pre-test, confirmatory factor analysis (CFA) was conducted, three items were deleted because of their low factor loadings. Based on the modification index, four items with low factor loads and high modification index values were deleted. After item refinement and confirmatory factor analysis (CFA), 13 items were retained across four dimensions of chemical epistemological beliefs: “Source (3 items:),” “Certainty (3 items:),” “Simplicity (4 items:),” and “Justification (3 items:).” After modification, the CEBQ (Appendix 1) model fit well: χ^2^/*df* = 1.112, standardized root mean square residual (SRMR) = 0.059, comparative fit index (CFI) = 0.985, Tucker–Lewis index (TLI) = 0.981, and root mean square error of approximation (RMSEA) = 0.036, and the factor loadings of all 13 items were within the range of 0.640–0.875.

#### 4.2.2 CT disposition

A measure of CT disposition was adapted from Zou et al. (2023). All items were presented on a Likert-type scale ranging from 1 (strongly disagree) to 7 (strongly agree). Higher average scores indicate a stronger tendency toward critical thinking disposition. The items included “When learning chemistry, I would evaluate different opinions to see which one is more reasonable”; “When learning chemistry, I try to understand what I have learned from different perspectives”; and “It is easier for me to agree with my friends when discussing chemistry.” One item—“lt is easier for me to agree with my friends when discussing chemistry”—was reverse-coded. That is, a response of 1 was converted to 7, 2–6 and so on. After confirmatory factor analysis (CFA), the hypothesized single-factor model with three items was saturated (degrees of freedom *df* = 0) because the number of estimated parameters equaled the number of unique elements in the covariance matrix. As a result, traditional fit indices (e.g., χ^2^, RMSEA, CFI, TLI) could not provide meaningful information. However, the standardized factor loadings were all statistically significant (λ = 0.690–0.874, *p* < 0.001), indicating a strong relationship between the items and the latent construct. The standardized root mean square residual (SRMR = 0.000) further suggested no discrepancy between the model-implied and observed covariance matrices, though this is an inherent feature of saturated models.

#### 4.2.3 Chemistry learning approaches

Chemistry learning approaches were adapted from the Scale for Approaches to Learning Chemistry, a self-report instrument developed by Li et al. (2013). Two questions about the depth and one question about the surface strategy. The items included “When I learn new contexts about chemistry, I try to explore the relationships with other contexts I learned before”; “I try to understand the meaning of the contents when I learn chemistry”; and “I will notice and memorize the parts when I learn chemistry instead of understanding it.” Each item was rated on a Seven-point Likert-type scale (1 = strongly disagree; 7 = strongly agree). The surface strategy item (Item 3) was reverse-coded (i.e., 1 was recoded as 7, 2 as 6,…, and 7 as 1). Higher average scores thus reflect stronger tendencies toward deep learning. An average score was computed after reverse-coding to reflect overall learning orientation, with higher values indicating deeper learning tendencies. Similarly, the single-factor saturated model of Chemistry Learning Approaches was tested. All standardized factor loadings demonstrated statistical significance (λ = 0.601–0.872, *p* < 0.001), confirming robust relationships between the measurement items and the latent construct.

#### 4.2.4 Chemistry disciplinary competence

The task and performance items used to assess chemistry disciplinary competence in this research were developed according to Wang's (2022) chemistry disciplinary competence standards, creating projects under the categories of Understanding, Applying, and Innovating to gauge students' disciplinary competence in chemistry. Most chemistry performance items were adapted from college entrance examination materials. We first conducted analysis and matching on the selected materials in accordance with the framework developed by Wang L. et al. (2022) for assessing high school students' chemistry disciplinary competence, ensuring that each item meets the requirements specified in the framework. For example, some examples of specific question designs that conform to each dimension of this framework are as follows:

- For the A1-Recognize dimension, the designed item is: “Please write the oxygen-containing functional groups in 2-(propan-2-yl) phenol (presented as a chemical structural formula in the original item).”

- For the B3-Design dimension, we modified items to align with this dimension, e.g., “Design an experimental method to test for the presence of a small amount of toluene in hexene.”

- For the C2-Predicting dimension, we developed an item requiring the writing of isomers for an unfamiliar organic compound, with the following requirements: the molecule contains a benzene ring; it can undergo a silver mirror reaction; there are three absorption peaks in the nuclear magnetic resonance hydrogen spectrum. The task is to write the isomers of 4-methoxy-3-(propan-2-yl) phenyl cyanomethyl ether (presented as a chemical structural formula in the original item).

The measure of chemistry disciplinary competence was validated through an expert review process and complemented by the Rasch measurement. Four experts—two professors of chemistry education and two experienced teachers specializing in chemistry teaching and research—evaluated the academic performance instrument. After the pre-test, the items were finally adjusted based on the results of the Rasch model. Ultimately, the assessment instrument for students' chemistry disciplinary competence took the form of a paper-and-pen test, consisting of 15 items with a total score of 15. Each item adopted a dichotomous scoring method, where 1 point was awarded for a correct answer and 0 points for an incorrect one.

### 4.3 Data analysis procedures and tools

Statistical analyses were conducted using SPSS 23.0 and Mplus 8.3. Specifically, SPSS 23.0 was used to perform descriptive statistics (means, SD) and Pearson correlations; Mplus 8.3 was employed to examine the mediating roles of critical thinking disposition and chemistry learning approaches between chemical epistemological beliefs and chemistry disciplinary competence.

## 5 Results

### 5.1 Suitability for measurement instruments

#### 5.1.1 Chemical epistemological beliefs

Following revisions based on the pre-test, the formal questionnaire demonstrated good reliability and validity. CFA results indicated that the modified questionnaire model maintained a good fit: χ^2^/*df* = 1.658; SRMR = 0.046; CFI = 0.962; TLI = 0.950; RMSEA = 0.060; the factor loadings of all 13 items were within the range of 0.592–0.874. The Cronbach's α for the entire questionnaire was 0.784.

#### 5.1.2 CT disposition and chemistry learning approaches

The Cronbach's α coefficients for the Critical Thinking Disposition Questionnaire and the Chemistry Learning Methods Questionnaire were 0.837 and 0.880, respectively, both exceeding the threshold of 0.7. The measurement models showed strong psychometric properties, with statistically significant standardized factor loadings ranging from 0.647 to 0.879 (*p* < 0.001) for critical thinking disposition and 0.787 to 0.885 (*p* < 0.001) for chemistry learning approaches. These values suggest that the questionnaires have satisfactory internal consistency, indicating good reliability.

#### 5.1.3 Chemistry disciplinary competence

Chemistry disciplinary competence was measured using a one-dimensional Rasch model. Rasch analysis was performed via Winstep 3.72.0 software to establish evidence of the validity and reliability of the data.

**Figure 2:**
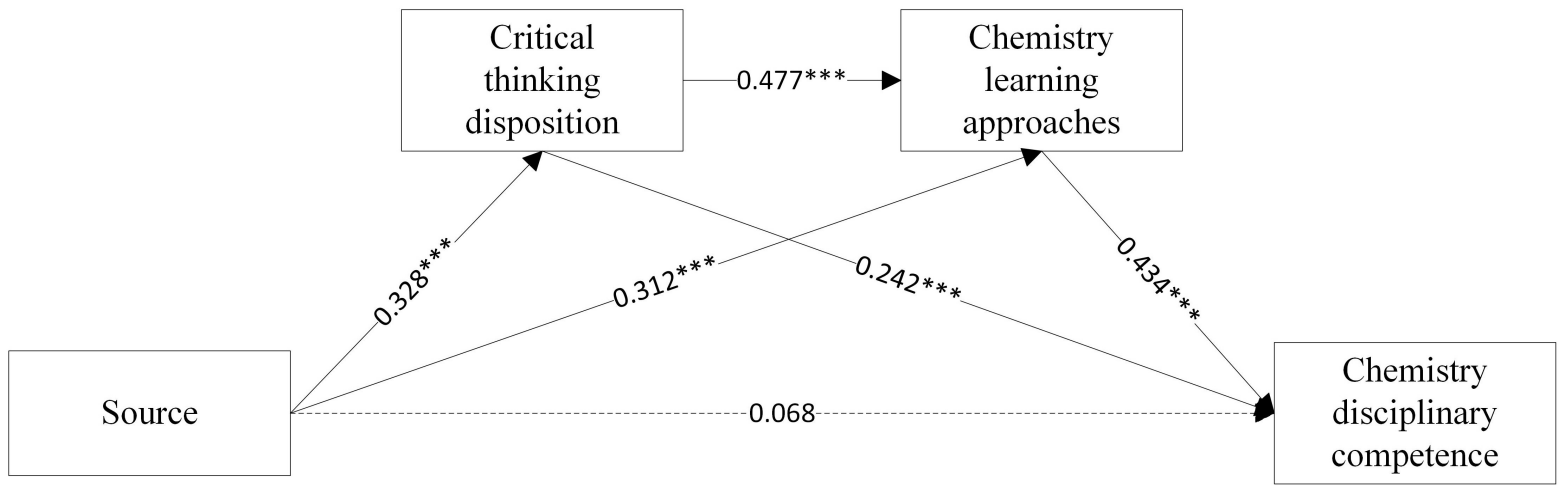
The chain mediating effect of Source beliefs and chemistry disciplinary competence.

After revision, the measure of chemistry disciplinary competence demonstrated good validity. The results indicated that person reliability was 0.83 and item reliability was 0.99 (Appendix 2, Figure 1). Both values surpassed the 0.7 benchmark, thereby fulfilling the reliability criteria for the diagnostic tests. The Wright map, which indicated the construct validity of the measures, showed that item difficulty aligned with students' abilities and covered their full range (Appendix 2, Figure 2). The variance in this study was 66.6%, which surpassed the 50% threshold required by the Rasch criteria, thereby supporting the unidimensionality of the scale. The unexplained variance in the first construct was 1.8, below the recommended value of 2 (Appendix 2, Figure 2), indicating that the instrument likely does not measure another construct.

**Figure 3:**
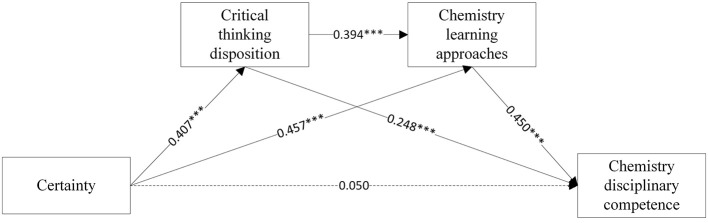
The chain mediating effect of Certainty beliefs and chemistry disciplinary competence.

The results of the one-dimensional test in Appendix 2, Figure 3 illustrate the factor loadings of the residuals. Among all 15 items, the INFIT MNSQ values ranged from 0.59 to 1.47 (refer to Appendix 2, Figure 3), all of which fall within the acceptable limits of 0.5 to 1.5. Factor analysis of the residuals demonstrated that almost all 18 items (except for three items: 8, 9, and 10, which represent the three items A, B, and C, respectively) had loadings (i.e., correlation) within the 0.4 to +0.4 range (Appendix 2, Figure 4). This range indicates a strong fit between the 15-item test and the model. In summary, the data satisfied the criteria of unidimensionality and local independence, thereby supporting the construct validity of the assessment tool.

### 5.2 Common method variance

Harman's single-factor test was performed to assess potential common method bias, given the cross-sectional measurement of chemical epistemological beliefs, critical thinking disposition, and chemistry learning approaches. The first extracted factor explained 31.415% of total variance, below the 40% benchmark, No statistically discernible common method bias was identified in the relationships between key variables through rigorous single-source diagnostics.

### 5.3 Descriptive statistics and bivariate correlations

Means, standard deviations and correlations between the research variables are presented in Table 2. As shown in the Table 2, within the dimensions of chemical epistemological beliefs: Source exhibited significant positive correlations with CT disposition (*r* = 0.328, *p* < 0.001), Chemistry learning approaches (*r* = 0.469, *p* < 0.001), and Chemistry disciplinary competence (*r* = 0.358, *p* < 0.001); Certainty demonstrated significant positive correlations with CT disposition (*r* = 0.407, *p* < 0.001), Chemistry learning approaches (*r* = 0.617, *p* < 0.001), and Chemistry disciplinary competence (*r* = 0.429, *p* < 0.001). Regarding Simplicity and Justification beliefs, Pearson correlation analysis showed that neither dimension was significantly correlated with CT critical thinking disposition, Chemistry learning approaches, nor Chemistry disciplinary competence (all *p* > 0.05). Given the absence of significant associations, these two dimensions were excluded from the subsequent SEM analysis, as the lack of bivariate correlations makes it difficult to establish a meaningful linear relationship in the structural model.

**Table 2:** Descriptive statistics and bivariate correlations.

**Measure**	**M**	**SD**	**1**	**2**	**3**	**4**	**5**	**6**	**7**	**8**
1. Gender	1.480	0.501	1							
2. Source	3.460	1.364	0.03	1						
3. Certainty	4.669	1.494	0.08	0.654^**^	1					
4. Simplicity	6.315	0.598	0.149^*^	0.205^**^	−0.154^*^	1				
5. Justification	5.806	1.039	0.018	0.145	0.117	0.273^**^	1			
6. CT disposition	5.117	1.233	0.07	0.328^**^	0.407^**^	−0.09	−0.025	1		
7. Chemistry learning approaches	4.612	1.329	0.07	0.469^**^	0.617^**^	−0.111	0.027	0.580^**^	1	
8. Chemistry disciplinary competence	0.990	2.453	0.002	0.358^**^	0.429^**^	−0.126	−0.073	0.530^**^	0.625^**^	1

### 5.4 The chain mediating effects analyses

The multiple linear regression results are showed in Table 3, Figures 2, 3. Firstly, Equations A1 and B1 showed that source (β = 0.328, *p* < 0.001) and certainty (β = 0.407, *p* < 0.001), respectively, exerted significant positive effects on CT disposition. Second, Equation A2 showed that source directly predicted chemistry learning approaches (β = 0.477, *p* < 0.001). With control applied on source beliefs, CT disposition further enhanced this relationship (β = 0.312, *p* < 0.001). In Equation B2, certainty significantly predicted learning approaches (β = 0.394, *p* < 0.001), with CT disposition maintaining its positive influence (β = 0.457, *p* < 0.001). Thirdly, upon incorporating chemistry disciplinary competence into the regression equation, both critical thinking disposition (β = 0.242, *p* < 0.001, Equation A3; β = 0.244, *p* < 0.001, Equation B3) and learning approaches (β = 0.434, *p* < 0.001, Equation A3; β = 0.434, *p* < 0.001, Equation B3) significantly contributed to chemistry disciplinary competence. Yet source (β = 0.068, *p* > 0.05) and certainty beliefs (β = 0.050, *p* > 0.05) did not directly predict chemistry disciplinary competence. The non-significant direct effects of source and certainty beliefs on disciplinary competence suggest their influence is fully mediated by CT disposition and learning approaches, aligning with Hofer (2000) domain-specific epistemology model where beliefs operate through metacognitive processes rather than directly shaping performance.

**Table 3:** Results of multiple linear regression.

**Model type**	**Outcome variable**	***R* ^2^**	**β**	***t***	**BOOTSTRAP 95% CI**	
					**Lower**	**Upper**
**Equation (A1)**						
Source	Disposition	0.107	0.328	5.236	0.193	0.442
**Equation (A2)**						
Source	Approach	0.423	0.477	5.312	0.196	0.426
Disposition			0.312	7.932	0.357	0.589
**Equation (A3)**						
Source	Competence	0.412	0.068	1.055	−0.057	0.194
Disposition			0.242	3.141	0.080	0.381
Approaches			0.434	5.869	0.285	0.580
**Equation (B1)**						
Certainty	Disposition	0.166	0.407	6.050	0.259	0.526
**Equation (B2)**						
Certainty	Approach	0.510	0.394	7.730	0.339	0.569
Disposition			0.457	6.187	0.268	0.516
**Equation (B3)**						
Certainty	Competence	0.410	0.050	0.661	−0.101	0.194
Disposition			0.244	3.140	0.083	0.385
Approaches			0.434	5.651	0.285	0.589

Then, Mplus 8.3 was used to verify whether the direct and indirect effects of chemical epistemological beliefs on chemistry disciplinary competence were significant. As the analytical results show in Table 4, the total effect of source beliefs on chemistry disciplinary competence was 0.351 (95% CI is 0.224–0.469); the total effect of certainty beliefs on chemistry disciplinary competence was 0.417 (95% CI is 0.299–0.532). Hypothesis 1 is supported. The total indirect effects of source (0.283, 95% CI is 0.197–0.383, accounting for 81%) and certainty (0.367,95% CI is 0.163-0.489, accounting for 88%) beliefs on chemistry disciplinary competence were significant. Specifically, chemical epistemological beliefs affect students' chemistry disciplinary competence via three indirect routes: the indirect effect of source → CT disposition → chemistry disciplinary competence was 0.079 (95% CI is 0.029–0.145), the indirect effect of certainty → CT disposition → chemistry disciplinary competence was 0.099 (95% CI is 0.036–0.185), supporting hypothesis 2. The indirect effect of source → chemistry learning approaches → chemistry disciplinary competence was 0.135 (95% CI is 0.078–0.216), the indirect effect of certainty → chemistry learning approaches → chemistry disciplinary competence was 0.198 (95% CI is 0.124–0.292), supporting hypothesis 3; The indirect effect of source → CT disposition → chemistry learning approaches → chemistry disciplinary competence was 0.068(95% CI is 0.033–0.120), the indirect effect of certainty → CT disposition → chemistry learning approaches → chemistry disciplinary competence was 0.069 (95% CI is 0.036–0.125), supporting hypothesis 4. The effects of the direct paths were insignificant, indicating that the mediating variable played a full mediating role.

**Table 4:** Indirect effect of critical thinking disposition and chemistry learning approaches.

**Model type**	**Effect**	**Boot SE**	**BOOTSTRAP 95% CI**		**Ratio of indirect to total effect (%)**
			**Lower**	**Upper**	
Total effect	0.351	0.064	0.224	0.469	
Direct effect	0.068	0.065	−0.057	0.194	
Total indirect effect	0.283	0.047	0.197	0.383	81
Indirect effect A1	0.079	0.029	0.029	0.145	23
Indirect effect A2	0.135	0.034	0.078	0.216	38
Indirect effect A3	0.068	0.022	0.033	0.120	19
Total effect	0.417	0.059	0.292	0.523	
Direct effect	0.050	0.076	−0.101	0.194	
Total indirect effect	0.367	0.058	0.263	0.489	88
Indirect effect B1	0.099	0.036	0.036	0.185	24
Indirect effect B2	0.198	0.043	0.124	0.292	47
Indirect effect B3	0.069	0.022	0.036	0.125	17

Indirect effect A1/B1 included source/certainty, critical thinking disposition, and chemistry disciplinary competence.

Indirect effect A2/B2 included source/certainty, chemistry learning approaches, and chemistry disciplinary competence.

Indirect effect A3/B3 included source/certainty, critical thinking disposition, chemistry learning approaches, and chemistry disciplinary competence.

## 6 Discussion and conclusion

### 6.1 The relationship between chemical epistemological beliefs and chemistry disciplinary competence

The primary contribution of this study is the identification of the dimensions of chemical epistemological beliefs that influence the development of chemistry disciplinary competence. Specifically, this study found that Certainty beliefs and Source beliefs exert a significant influence on chemistry disciplinary competence, while Justification beliefs and Simplicity beliefs did not exhibit statistical significance.

As for Certainty beliefs, which our study identified as a dimension exerting significant influence on chemistry disciplinary competence, many theories and much evidence have directly or indirectly suggested certainty beliefs can significantly predict chemistry disciplinary competence. For example, (Trautwein and Lüdtke 2007) found certainty beliefs to be significant predictors of final school grades in a large-scale study. Students who acknowledge the evolving nature of scientific knowledge are more likely to investigate alternative explanations, leading to a more nuanced and comprehensive understanding of the subject matter (Aditomo, 2018). However, students who believe that their knowledge is stable find it difficult to engage in in-depth information processing (Trautwein and Lüdtke, 2007). Collectively, these findings from existing literature align with our study's conclusion, highlighting that Certainty beliefs shape chemistry disciplinary competence by influencing students' perceptions of knowledge dynamics and their subsequent information-processing behaviors—reinforcing the critical role of this dimension in chemistry learning and competence development.

Within the Source dimension, although some studies have focused on the relationship between source beliefs and students' academic performance, the relationship has been unclear. For instance, Özbay and Köksal's (2021) study found that the “source” aspect negatively predicted science achievement while other aspects of scientific epistemological beliefs positively predicted science achievement. Hofer (2000) and Aditomo (2018) found that source beliefs did not predict students' grades. Many examinations are assessed based on the consistency of students' views and understanding of the normative knowledge taught by teachers (Aditomo, 2018). Memorizing certain facts and learning from authorities can also improve grades (Özbay and Köksal, 2021). Therefore, even students with immature source beliefs can perform better in exams through memory. In this study, disciplinary competence provides a more comprehensive description of student development, avoiding measurement errors caused by overly simple test questions. We found that complex source beliefs can positively predict students' chemistry disciplinary competence. However, those who hold mature source beliefs perceive that chemical knowledge is actively constructed by learners through reasoning, experimentation, and evidence evaluation, rather than passively received from authority. Such students are more likely to have confidence in their own abilities, learn for curiosity and mastery (Alpaslan, 2017), and understand and see science in a new way without simply memorizing (Liang and Tsai, 2010). Students who regard authorities as exclusive sources of knowledge are less inclined to embrace intellectual risk-taking (Özbay and Köksal, 2021), potentially stifling their creativity. Wan et al. (2021) found that complex source beliefs have a positive impact on STEM creativity. Therefore, the effect of source beliefs on students' chemistry disciplinary competence may be reflected more in innovating competence. To summarize, existing studies on the relationship between source beliefs and academic outcomes have been inconsistent, largely because traditional academic performance measurements (e.g., exam grades) rely heavily on memory of normative knowledge and consistency with teacher-taught content—an approach that masks the true role of source beliefs in supporting students' comprehensive development. This is where the key contribution of our study lies: by adopting chemistry disciplinary competence as the outcome variable, we not only resolved the ambiguity in previous research but also clearly identified that complex, mature source beliefs exert a positive predictive effect on chemistry disciplinary competence. Furthermore, we linked this effect to the development of innovating competence, revealing that mature source beliefs foster creativity and in-depth learning behaviors (e.g., active knowledge construction, intellectual risk-taking) that are critical to disciplinary competence. This finding fills the research gap in understanding how source beliefs influence chemistry-specific comprehensive abilities, provides empirical evidence for the value of targeting source belief development in chemistry education, and highlights the importance of using appropriate outcome measures to capture the true impact of epistemological beliefs.

Simplicity and Justification showed no statistically significant correlations with chemistry disciplinary competence. Prior research suggests that the knowledge structure and epistemological assumptions vary across disciplines, and the characteristics of a specific discipline may reduce the explanatory power of certain epistemological dimensions (Hofer, 2000). Epistemological beliefs may be influenced by disciplinary norms, which could weaken their statistical significance (Aditomo, 2018). Chemistry, as a highly structured discipline (Talanquer, 2011), requires students to integrate knowledge networks across multiple representational levels (macroscopic, submicroscopic, and symbolic). Moreover, the view of chemistry as an experimental natural science has been consistently emphasized from students' first exposure to the subject. In the Chinese high school context, a standardized curriculum, unified textbooks, and an examination—oriented pedagogy further promote homogeneity in students' beliefs about the complexity of knowledge and the justification of chemical knowledge. As shown in the descriptive statistics in Table 2, compared to other epistemological constructs (e.g., Source: SD = 1.364; Certainty: SD = 1.494), the Simplicity (M = 6.315, SD = 0.598) and Justification (M = 5.806, SD = 1.039) dimensions exhibited relatively higher mean scores but lower standard deviations, indicating a high degree of homogeneity in students' Simplicity and Justification beliefs. It is also possible that students' high scores in these two dimensions reflect responses shaped by classroom instruction rather than genuinely internalized epistemological beliefs. Under such conditions, sophisticated epistemological stances regarding Simplicity and justification may have limited opportunities to be applied in classroom learning and assessment, resulting in insufficient individual differences to exert a measurable effect on chemistry disciplinary competence. Future researchers could first develop chemistry-specific measurement tools for Simplicity and Justification beliefs and expand sample contexts to better capture individual differences in these beliefs. They could also design targeted instructional interventions to reduce belief homogeneity and explore whether such interventions help reveal the potential links between these two dimensions and chemistry disciplinary competence.

### 6.2 Mediating roles of CT disposition and chemistry learning approaches

The second contribution of this study lies in its exploration of the mediating effects of CT disposition and chemistry learning approaches on the relationship between chemical epistemological beliefs and chemistry disciplinary competence. The indirect effects between chemical epistemological beliefs and chemistry disciplinary competence were all significant and the direct effect was insignificant suggesting that both chemistry learning burnout and chemistry learning flow played total mediating roles in the relationship between metacognition and chemistry identity.

As posited by the Reflective Judgment Model (King and Kitchener, 1994), learners' epistemological beliefs—such as whether knowledge is static or evolving, whether it is provided by an authority—serve as a foundational framework for engaging in critical evaluation and logical reasoning. These beliefs create cognitive readiness to question assumptions, analyze evidence, and synthesize interdisciplinary perspectives, which collectively define CT disposition (Facione et al., 2000b). This implies that students with sophisticated chemical epistemological beliefs are more likely to adopt a proactive CT disposition. Consequently, the relationship between chemical epistemological beliefs and chemistry disciplinary competence may be mediated by CT disposition: epistemological beliefs shape students' inclination to critically evaluate chemical phenomena, while such dispositional tendencies directly enhance their ability to understand knowledge reflectively and solve complicated problems which are key points of disciplinary competence. This result is in accordance with the study of Talanquer (2013).

According to the 3P Model of Learning (Biggs, 1987), students' epistemological beliefs, as a presage factor, fundamentally shape their learning motivations and strategies. Crucially, deep learning approaches help cultivate students' higher-order thinking skills and enhance their ability to internalize complex disciplinary competence (Ye and Xu, 2023), enabling them to apply knowledge critically and solve problems creatively (Jiang, 2022). This mediation mechanism is supported by Learning Patterns Theory (Vermunt, 1996). Students with advanced epistemological beliefs tend to adopt deep learning strategies that help them develop advanced problem-solving skills and retain knowledge longer, ultimately boosting their disciplinary competence.

### 6.3 The chain mediating effect of CT disposition and chemistry learning approaches

As hypothesized, the chain mediating effect of chemical epistemological beliefs → CT disposition → chemistry learning approaches → chemistry disciplinary competence was significant. This finding suggests that CT disposition mediates the relationship between epistemological beliefs and students' choice of learning strategies, whereas chemistry learning approaches serve as a pathway through which CT disposition enhances chemistry disciplinary competence. This finding aligns with previous research suggesting that students who hold more sophisticated epistemological beliefs are more likely to develop a stronger critical thinking disposition (Greene and Yu, 2016; Muis et al., 2021). Such a disposition enables students to select deeper and more self-regulated learning approaches (Kusmaryono and Nizaruddin, 2023), which have been found to positively impact their disciplinary understanding and ability to solve complex problems (Fawzia and Karim, 2024; Nelson Laird et al., 2008). Furthermore, students engaging in deep learning approaches tend to construct knowledge meaningfully, integrate new and existing knowledge, and apply concepts to novel situations which are all core components of disciplinary competence in chemistry (Talanquer, 2013; Tariq et al., 2025).

## 7 Conclusion and recommendations for practice

Research has demonstrated that Chinese students tend to hold less sophisticated source beliefs and certainty beliefs—two core dimensions of chemical epistemological beliefs. Yet these beliefs exert a significant influence on students' learning: they first shape students' CT disposition and chemistry learning approaches, which then further impact the development of students' chemistry disciplinary competence.

In the context of Chinese education, students' development of source beliefs and certainty beliefs often faces challenges arising from specific cultural and instructional traditions. The collectivist cultural tradition emphasizes respect for authority and the maintenance of consensus and harmony, which, when reflected in education, tends to foster a teacher- and textbook-centered knowledge transmission model. This approach may somewhat suppress students' critical reflection on the sources of knowledge and its uncertainties. “Chinese students' underperformance in source beliefs and certainty beliefs can be attributed to a complex interplay of cultural and systemic factors. While the traditional Confucian norm of “once a teacher, always a father” underscores a deep respect for authority, this phenomenon is more comprehensively understood through the broader lens of collectivism, which permeates Chinese society and its educational system (Wang, 2023). Collectivist cultures prioritize group harmony, consensus, and adherence to established norms. In the classroom, this translates into a strong emphasis on conformity and the acceptance of knowledge from authoritative sources (teachers and textbooks) as a way to maintain social cohesion and avoid conflict. They learn to rely on authoritative sources not solely out of reverence, but as a pragmatic strategy for success within a system that collectively defines goals and paths to achievement. This collective orientation, while fostering discipline and respect, can inadvertently suppress the individual epistemic agency needed to question sources, tolerate ambiguity, and view knowledge as dynamic. Therefore, it is the synergy between specific Confucian values and the overarching collectivist structure that shapes a learning environment where critical thinking disposition and deep conceptual learning in subjects like chemistry may be challenged.

Second, China's education system is famously exam-driven This examination-oriented culture permeates all levels of schooling and prioritizes content mastery and performance on standardized tests. As Yan (2015) observes, even well-intentioned curricular reforms face “exam culture” backwash – teachers and students feel compelled to focus on tested knowledge at the expense of inquiry. This systemic focus on high-stakes exams reinforces the belief that knowledge is a fixed set of facts to be learned for the test. The educational assessment system rewards students for producing “standardized curriculum” knowledge and “‘correct' answers” rather than for asking questions or exploring uncertainties. As a result, students internalize a pragmatic approach: they rely on authoritative sources (textbooks, teachers) and seek certain answers to succeed in exams. Over time, this habituation fosters the epistemic view that knowledge is certain and comes from authority, since those are the notions that yield rewards in the system. In a large-scale survey of Chinese secondary students, Zhang and Ding (2013) found that more years of traditional lecture-based instruction (within this exam-oriented system) led to less expert-like epistemological beliefs about science. In other words, as students progressed through the grades, their views of knowledge became increasingly absolutist—a likely outcome of years of curriculum emphasizing content acquisition over critical inquiry. By Grade 12, many students were less inclined to see knowledge as evolving or to view learning as conceptually driven, reflecting the cumulative impact of an exam-focused curriculum. The educational assessment system further reinforces this by rewarding the mastery of a standardized curriculum and “correct” answers, rather than open-ended inquiry. Consequently, students may internalize a learning approach that values efficiency and certainty over critical examination. In addition, classroom pedagogy in China has traditionally been teacher-centered, characterized by didactic instruction and rote learning (Wang, 2011). Teachers are viewed as the primary source of knowledge, and students are expected to listen and absorb. In science education, this translates into an emphasis on factual recall and following set procedures rather than open-ended exploration. This didactic pedagogy thus also perpetuates certainty and source beliefs.

The nature of science reveals that scientific theories are not immutable truths but constantly evolve through an interplay of truth and fallacy (Brock and Park, 2024; McComas, 1998). Without an understanding of the historical development of scientific models, students can easily develop rigid epistemic beliefs, regarding knowledge as static and derived from unchallengeable authorities. Teachers can utilize the history of science in class to provide students with a deeper understanding of the nature of science, including the provisional and developmental aspects of scientific knowledge, thereby helping students to establish mature epistemological beliefs. For example, when teaching about atomic structure models, teachers should emphasize how scientific models evolve over time and with scientific advancements, as well as how each model is modified or replaced based on new experimental data and theoretical developments (Justi and Gilbert, 2000).

In addition to using historical case studies, teachers can promote students' epistemological beliefs by engaging them in inquiry-based and argumentation-oriented activities. Research shows that when students are encouraged to ask questions, design investigations, and construct explanations, they are more likely to view knowledge as tentative and evidence-based rather than absolute (Patterson and Ding, 2025; Tan et al., 2023). Classroom argumentation, in particular, enables students to critically evaluate claims, justify reasoning with evidence, and appreciate the fallibility of knowledge (Osborne et al., 2004). These practices are especially valuable in Chinese classrooms where teacher-centered instruction often dominates, as they provide opportunities for students to shift from passive acceptance of authority toward active engagement with sources of knowledge.

Finally, providing professional development for teachers is essential. Research indicates that teachers' own epistemological beliefs strongly shape their instructional practices (Park et al., 2022; Wang H.-H. et al., 2022). Training that equips teachers with strategies to embed the Nature of Science (NOS), inquiry, and argumentation in their classrooms is crucial for effectively enhancing students' epistemological beliefs.

## 8 Limitations

This study focuses on students in 11th grade in high school, aiming to explore the potential influencing mechanisms between chemical epistemological beliefs and chemistry disciplinary competence. However, the study has several limitations that require further investigation in future research. First, it adopts a cross-sectional design, which can only reveal the correlations and possible causal relationships among variables, and cannot establish a definite causal link. Therefore, future studies should employ longitudinal methods to verify the causal relationships among these variables.

Second, this study's sample is limited to students in the eastern region of China, reflecting only the relationship between chemical epistemological beliefs and the formation of chemical disciplinary competence among students in this area. To enhance the generalisability and representativeness of the research findings, future studies should extend to student populations at different developmental stages and from different cultural backgrounds. This study is the relatively small sample size (*n* = 182), which may affect the generalizability of the findings. Although the sample size is within an acceptable range for structural equation modeling, a larger and more diverse sample would enhance the robustness and external validity of the results. Future research should replicate the study with broader populations to further validate the proposed model.

Third, the study relies on self-reported data when measuring chemical epistemological beliefs, critical thinking disposition, and chemical learning approach. Although self-reports can reflect individual subjective realities, some students may be more inclined to answer questions that they find acceptable rather than being entirely truthful. Therefore, future studies should collect data from students, parents, and teachers simultaneously. The results from these three sources can then be compared, further strengthening the validity and reliability of the research findings.

## Data Availability

The raw data supporting the conclusions of this article will be made available by the authors, without undue reservation.
