# Supplementary material for: How do chemical epistemological beliefs affect Chinese students' chemistry disciplinary competence? A structural equation modeling analysis
Source: Front Psychol. 2025 Nov 11;16:1599442. doi: 10.3389/fpsyg.2025.1599442 (PMC12643997; doi:10.3389/fpsyg.2025.1599442)
Supplement: Supplementary file 1 [file Supplementary_file_1.docx]

Supplementary Material

**Appendix 1**

# Chemical Epistemological Beliefs Questionnaire

## Source

The chemistry knowledge imparted by the teacher during class is entirely accurate.

The content in chemistry books can be confirmed as true.

Everyone must trust what chemists say.

## Certainty

There is only one right answer to every problem in chemistry.

The conclusions drawn by chemists through experimental observations are the only correct ones.

Chemical equations, theorems, and knowledge are eternal and unchanging.

## Justification

In chemical research, chemists use more than one method to test their hypotheses.

After numerous experiments, chemists can be more certain of the conclusions they have reached.

Chemical knowledge that stands up to experimental or practical testing is the only valid knowledge.

## Simplicity

Chemistry has little connection with other disciplines.

The chemistry learned in school has little use in everyday life.

The structure of substances I’ve learned has little to do with writing chemical equations.

The organic part has little connection with the rest of the chemistry content.

**Table1. Reliability and Convergent Validity of the Revised Chemistry Epistemological Beliefs Questionnaire**

| Dimension | Item | Estimate | S.E. | Z | P-Value | SMC | CR | AVE |
| --- | --- | --- | --- | --- | --- | --- | --- | --- |
| Source | SR1 | 0.776 | 0.071 | 10.966 | *** | 0.602 | 0.769 | 0.528 |
|  | SR2 | 0.648 | 0.081 | 7.958 | *** | 0.420 |  |  |
|  | SR3 | 0.749 | 0.072 | 10.466 | *** | 0.561 |  |  |
| Certainty | CR1 | 0.708 | 0.068 | 10.428 | *** | 0.501 | 0.773 | 0.533 |
|  | CR2 | 0.804 | 0.060 | 13.499 | *** | 0.646 |  |  |
|  | CR3 | 0.672 | 0.073 | 9.244 | *** | 0.452 |  |  |
| Justification | JS1 | 0.835 | 0.061 | 13.699 | *** | 0.697 | 0.813 | 0.593 |
|  | JS2 | 0.744 | 0.067 | 11.159 | *** | 0.554 |  |  |
|  | JS3 | 0.727 | 0.068 | 10.767 | *** | 0.529 |  |  |
| Simplicity | SP1 | 0.640 | 0.072 | 8.937 | *** | 0.410 | 0.860 | 0.608 |
|  | SP2 | 0.759 | 0.054 | 13.995 | *** | 0.576 |  |  |
|  | SP3 | 0.827 | 0.045 | 18.488 | *** | 0.684 |  |  |
|  | SP4 | 0.875 | 0.040 | 22.140 | *** | 0.766 |  |  |

**Appendix 2**


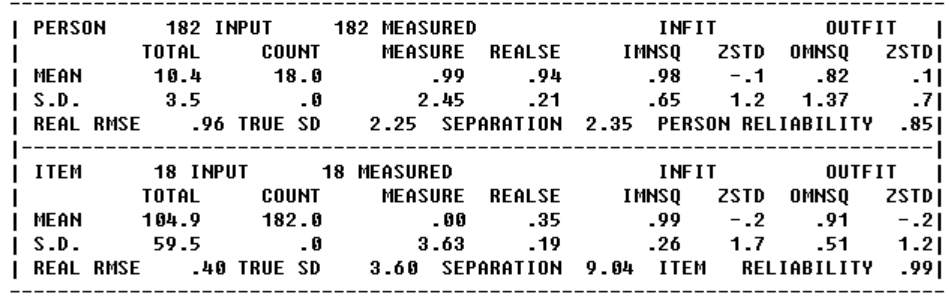


Figure 1. Reliabilities for the competence instrument


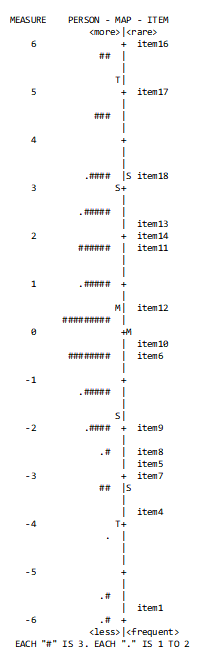


Figure 2. Wright map for the competence instrument


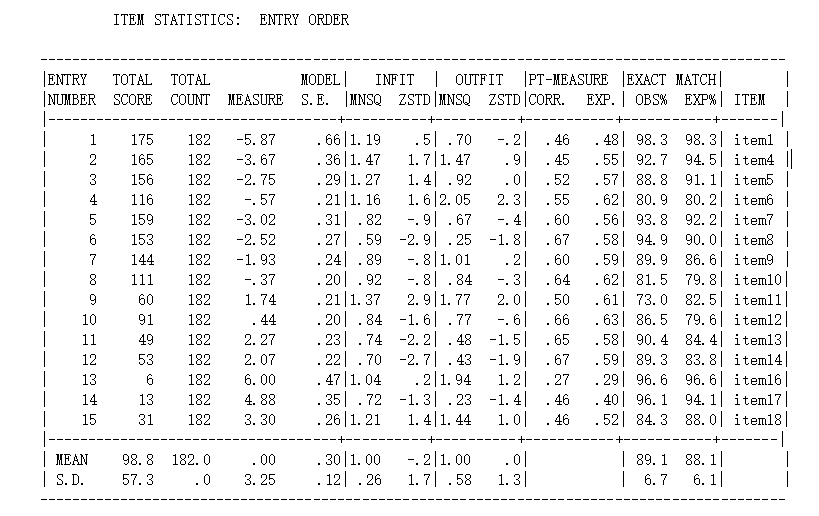


Figure 3. Item fit for the competence instrument


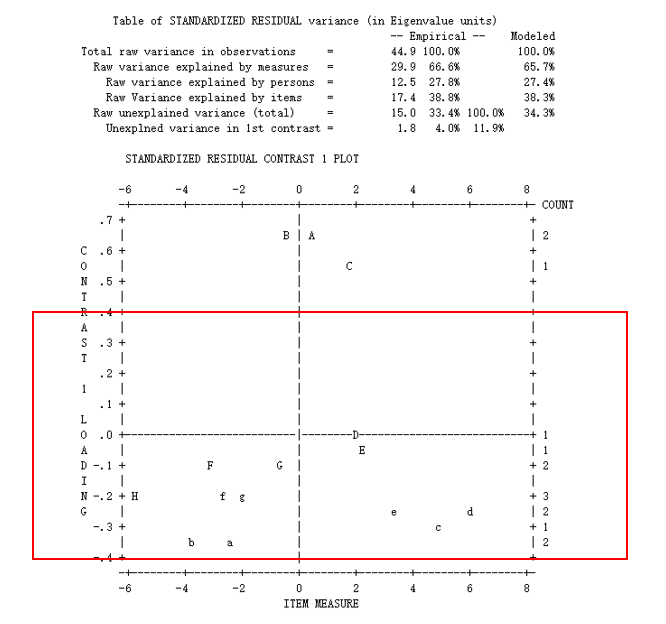


Figure 4. Construct 1 from principal component analysis
